# *G_m_*-Realization of Controlled-Gain Current Follower Transconductance Amplifier

**DOI:** 10.1155/2013/201565

**Published:** 2013-11-28

**Authors:** Worapong Tangsrirat

**Affiliations:** Faculty of Engineering, King Mongkut's Institute of Technology Ladkrabang (KMITL), Bangkok 10520, Thailand

## Abstract

This paper describes the conception of the current follower transconductance amplifier (CFTA) with electronically and linearly current tunable. The newly modified element is realized based on the use of transconductance cells (*G*_*m*_ *s*) as core circuits. The advantage of this element is that the current transfer ratios (*i*_*z*_/*i*_*p*_ and *i*_*x*_/*i*_*z*_) can be tuned electronically and linearly by adjusting external DC bias currents. The circuit is designed and analyzed in 0.35 **μ**m TSMC CMOS technology. Simulation results for the circuit with ±1.25 V supply voltages show that it consumes only 0.43 mw quiescent power with 70 MHz bandwidth. As an application example, a current-mode KHN biquad filter is designed and simulated.

## 1. Introduction

**Figure 1:**
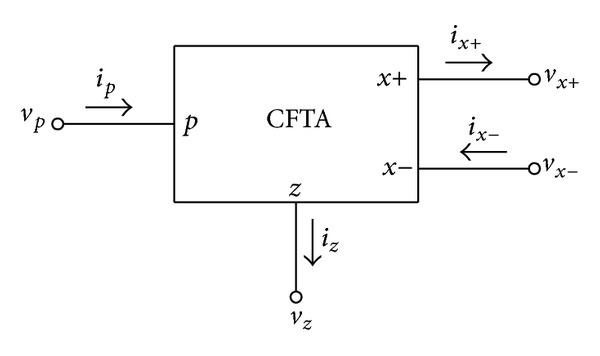
Schematic symbol of the CFTA.

Since its introduction in 2003, the current differencing transconductance amplifier (CDTA) has been proven to be a versatile active building block for current-mode signal processing circuits [1]. A large number of analog signal processing/signal generation circuit solutions based on this device have already been presented in the technical literature [1–12]. However, some earlier circuits described in [6–12] do not fully use the potential of the CDTAs, since always one of the input terminals *p* or *n* is not used. This may cause some noise injection into the monolithic circuit. Moreover, the current differencing property at an input stage can be achieved by the current feedback connection via the ±*x* terminals. This implies that the input terminals *p* or *n* are not necessarily required for some applications. In order to alleviate the mentioned problems, the modified version of the CDTA so-called current follower transconductance amplifier (CFTA) was recently introduced [13, 14]. The CFTA is a simplified variant of the CDTA element by replacing the current differencing unit with the simple current follower. Thus, this element can be thought of as a combination of the current follower, the current mirror, and the multioutput operational transconductance amplifier. As a result, a number of researches were published on CFTA-based applications [14–18]. As shown in Figure 1, the terminal relations of an ideal CFTA with its schematic symbol can be described by the following expression:

(1) $$\begin{array}{c} i_{z}=i_{p},\;\;i_{x+}=i_{x-}=g_{m}v_{z}=g_{m}Z_{z}i_{z}, \end{array}$$

where *g*_*m*_ is the transconductance gain of the CFTA and *Z*_*z*_ is an external impedance connected to the *z*-terminal. From (1), it can be seen that an ideal CFTA has a unity small-signal current transfer ratio (*α* = 1) between *i*_*z*_ and *i*_*p*_. In fact, for some applications such as automatic gain control circuit and tunable active filters, one wishes to alter the circuit transfer characteristics by electronic means. Thus, the usefulness of the CFTA is clearly an advantage to be gained, if *α* can be controlled electronically instead of a fixed one, in which case a more generalized CFTA is obtained. In the following, we have named the introduced device as the controlled-gain current follower transconductance amplifier (CG-CFTA).

In this paper, we present a generalized approach based on only transconductance (*G*_*m*_) elements for designing a CFTA with linearly and electronically current controllable. The proposed circuit consumes average power of 0.43 mW at ±1.25 V supply voltages. It operates as a linear circuit element and has a 3 dB bandwidth up to 70 MHz for 0.35 *μ*m TSMC CMOS technology. As an application example, a current-mode KHN-equivalent universal filter topology is implemented adopting the proposed circuit. PSPICE simulation results confirm the expected performances of the proposed circuit and its application in terms of linearity, tenability, and time and frequency responses.

## 2. Proposed Circuit Realization

In the following subsection, an approach to implement the proposed CG-CFTA using only tunable *G*_*m*_ elements is described, and its performance is also discussed.

### 2.1. Basic *G*_*m*_ Cell

**Figure 2:**
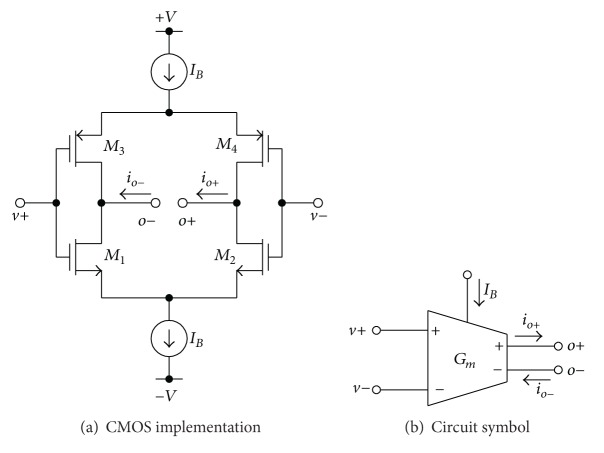
Basic *G*_*m*_ cell.

A particularly simple CMOS realization and the symbol of the tunable *G*_*m*_ cell, which will be used as a fundamental circuit for implementing the proposed circuit, are shown in Figures 2(a) and 2(b), respectively. The circuit is mainly composed of two Arbel-Goldminz transconductances [19]. For this element, the value of the transconductance (*G*_*m*_) can be determined by the output transistor transconductance, which can be approximated as

(2) $$\begin{array}{c} G_{m}≅\left(\frac{g_{m1}g_{m2}}{g_{m1}+g_{m2}}\right)+\left(\frac{g_{m3}g_{m4}}{g_{m3}+g_{m4}}\right), \end{array}$$

where $g_{mi}=\sqrt{K_{n(p)}I_{B}}\;(i=1,2,3,4)$ and *K*_*n*(*p*)_ is the transconductance parameter of NMOS (PMOS) transistor, and *I*_*B*_ is an external DC bias current of this element, respectively. Note that in (2) the value of *G*_*m*_ is electronically tunable by changing the bias current *I*_*B*_.

### 2.2. Linearly Tunable Current Amplifier

**Figure 3:**
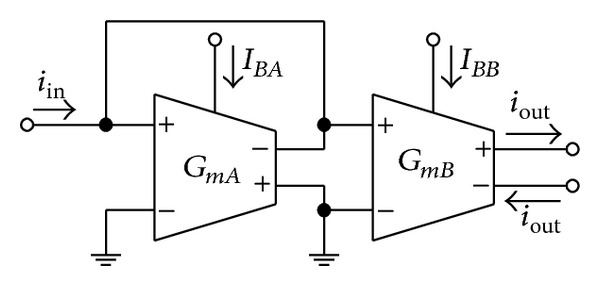
Current-controlled current amplifier (CCCA).

In Figure 3, the current-controlled current amplifier (CCCA) is realized by employing two *G*_*m*_ cells of Figure 2. In this case, the current gain of this circuit is given by

(3) $$\begin{array}{c} \alpha =\frac{i_{\text{out}}}{i_{\text{in}}}=\frac{G_{mB}}{G_{mA}}≅\sqrt{\frac{I_{BB}}{I_{BA}}}. \end{array}$$

**Figure 4:**
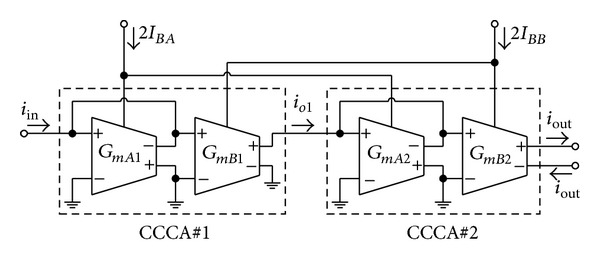
Linearly tunable current amplifier (LT-CA).

This expression shows that the current gain *α* is determined by the current ratio *I*_*BB*_/*I*_*BA*_, but it is in the form of square-root function. To alleviate this nonlinearity, we intend to realize the current amplifier that is possible to tune its gain linearly. In Figure 4, a possible realization of linearly tunable small-signal current amplifier is given, which is simply obtained from the cascade connection of two CCCAs in Figure 3. We also name this scheme as linearly tunable current amplifier (LT-CA). Hence, the current gain of this circuit can be found as follows:

(4) $$\begin{array}{c} \alpha =\frac{i_{\text{out}}}{i_{\text{in}}}=\frac{G_{mB1}G_{mB2}}{G_{mA1}G_{mA2}}≅\frac{I_{BB}}{I_{BA}}. \end{array}$$

Equation (4) clearly indicates that the current gain *α* of Figure 4 can be tuned electronically and linearly by means of the current ratio *I*_*BB*_/*I*_*BA*_. Furthermore, this cell will be used to design the input stage of the proposed CG-CFTA.

### 2.3. Linearly Tunable Transconductance Amplifier

**Figure 5:**
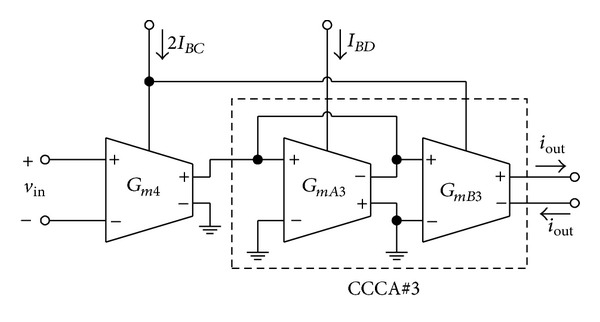
Linearly tunable transconductance amplifier (LT-TA).

Using the tunable *G*_*m*_ cell of Figure 2 and the CCCA of Figure 3, the electronically and linearly tunable transconductance amplifier (LT-TA) can be realized as shown in Figure 5. The output current (*i*_out_) of Figure 5 can be expressed as

(5) $$\begin{array}{c} i_{\text{out}}=\frac{G_{m4}G_{mB3}}{G_{mA3}}v_{\text{in}}. \end{array}$$

Substituting (2) into (5), the current *i*_out_ can be rewritten as

(6) $$\begin{array}{c} i_{\text{out}}=\left[\frac{I_{BC}\left(\sqrt{K_{n}}+\sqrt{K_{p}}\right)}{2\sqrt{I_{BD}}}\right]v_{\text{in}}=G_{mT}v_{\text{in}}, \end{array}$$

where *G*_*mT*_ denotes the transconductance gain of the LT-TA and can be expressed as

(7) $$\begin{array}{c} G_{mT}=I_{BC}K_{T}, \end{array}$$

(8) $$\begin{array}{c} K_{T}=\frac{\left(\sqrt{K_{n}}+\sqrt{K_{p}}\right)}{2\sqrt{I_{BD}}}, \end{array}$$

which can usually be kept at constant. It is clear from (7) that the *G*_*mT*_-value of LT-TA in Figure 5 can be electronically and linearly tuned by the bias current *I*_*BC*_.

## 3. Proposed CG-CFTA with Linearly Current Tunable

**Figure 6:**
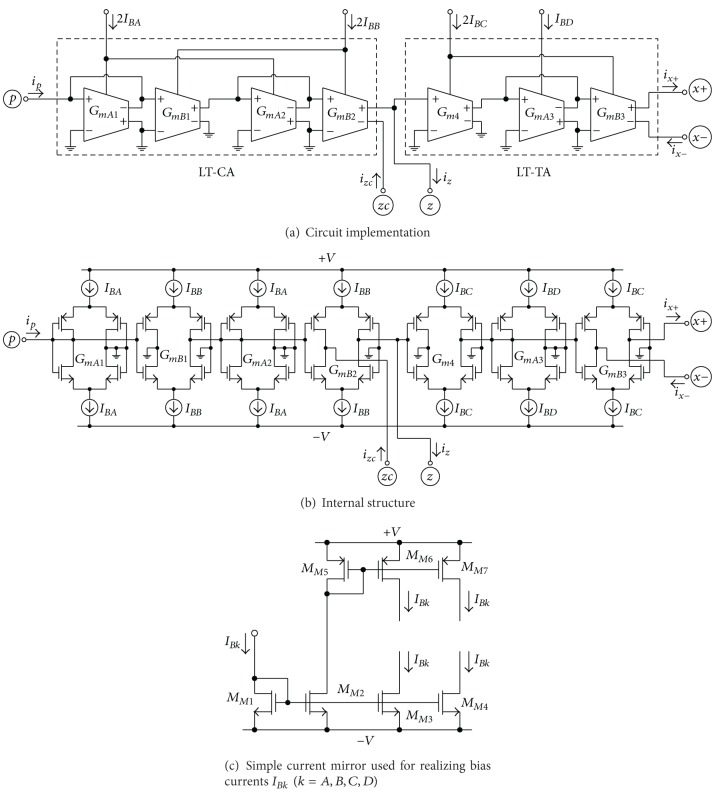
Proposed CG-CFTA.

**Figure 7:**
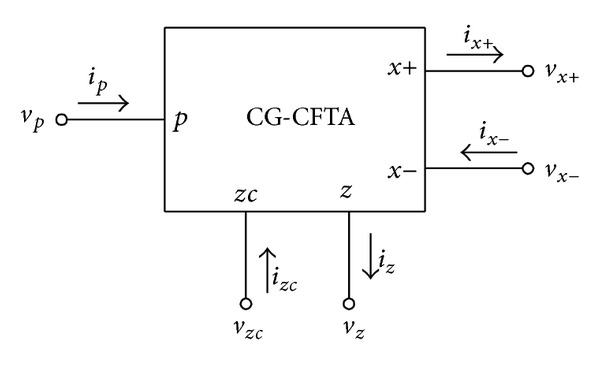
Circuit symbol of the proposed CG-CFTA.

The circuit implementation and internal structure of the proposed linearly tunable CG-CFTA is shown in Figures 6(a) and 6(b), respectively. It should be noted that the proposed circuit configuration is simply realized by the combination of the LT-CA from Figure 4, which is the input stage of the designed element, and the LT-TA from Figure 5, which functions as the output stage. For this realization, all the biasing currents (*I*_*BA*_, *I*_*BB*_, *I*_*BC*_, and *I*_*BD*_) were performed by the simple CMOS current mirrors as depicted in Figure 6(c). The input current flowing through the *p*-terminal (*i*_*p*_) is then reproduced on the *z*-terminal (*i*_*z*_) by two CCCAs with linear controlled gain, in such a way that *i*_*z*_ = *αi*_*p*_. In addition, the copy of the current *i*_*z*_ is also available at the *zc*-terminal (*i*_*zc*_). The voltage drop at the *z*-terminal (*v*_*z*_) is converted into output currents *i*_*x*+_ and *i*_*x*−_ by *G*_*mT*_-parameter of the LT-TA, which flows into output terminals *x*+ and *x*−. From the basic operation of the proposed CG-CFTA, the voltage-current characteristic of this device can be expressed as

(9) $$\begin{array}{c} i_{zc}=i_{z}={\alpha i}_{p},\;\;i_{x+}=i_{x-}=G_{mT}v_{z}=G_{mT}Z_{z}i_{z}, \end{array}$$

where its circuit symbol is represented in Figure 7.

As compared with the conventional CFTA, the designed CG-CFTA element has an advantage of providing a linear electronic controllability of the current transfer ratios *i*_*z*_/*i*_*p*_,  *i*_*zc*_/*i*_*p*_,  *i*_*x*+_/*i*_*z*_ and *i*_*x*−_/*i*_*z*_ via the following parameters: *α* ( = *I*_*BB*_/*I*_*BA*_) and *G*_*mT*_ ( = *I*_*BC*_ *K*_*T*_), respectively. As a consequence, it can be an advantage in a number of applications.

## 4. Simulation Results

The performance of the proposed CG-CFTA in Figure 6 has been verified by PSPICE simulation results. The simulation results were obtained using TSMC 0.35-*μ*m CMOS process model parameters. The dimensions *W*(*μ*m)/*L*(*μ*m) of the MOS transistors are set to be 56/0.7 for *M*_1_-*M*_2_, 32/0.7 for *M*_3_-*M*_4_, 7/0.7 for *M*_*M*1_, *M*_*M*5_–*M*_*M*7_, and 8.5/0.7 for *M*_*M*2_–*M*_*M*4_. The supply voltages used for the CG-CFTA are +*V* = −*V* = 1.25 V. The simulations are obtained for the following two configurations, that is, current transfer configuration (input current on *p* and outputs on *z* and *zc*) with *z* and *zc* grounded, and voltage-to-current configuration (input voltage on *z* and output currents on *x*+ and *x*−) with *x*+ and *x*− grounded.

**Figure 8:**
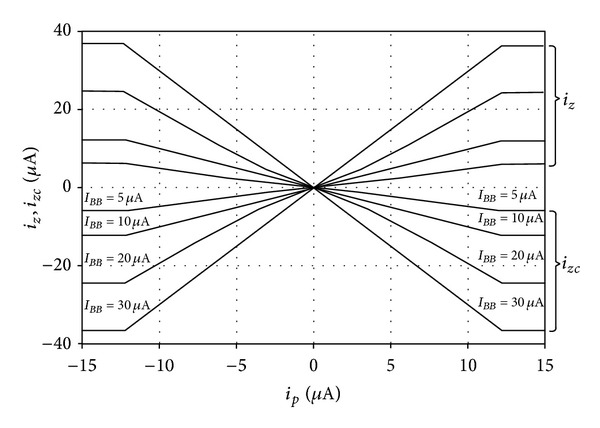
*i*_*z*_,  *i*_*zc*_ − *i*_*p*_DC transfer characteristics of the proposed CG-CFTA for different values of *I*_*BB*_.

**Figure 9:**
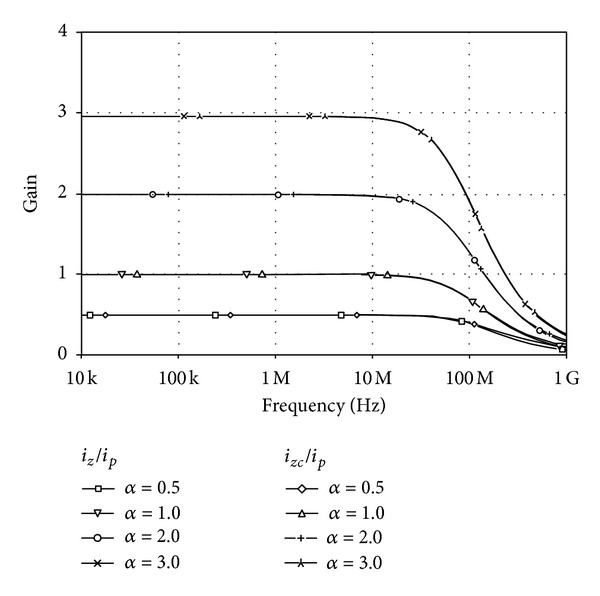
Frequency responses of *i*_*z*_/*i*_*p*_ and *i*_*zc*_/*i*_*p*_ for different values of *I*_*BB*_.

**Figure 10:**
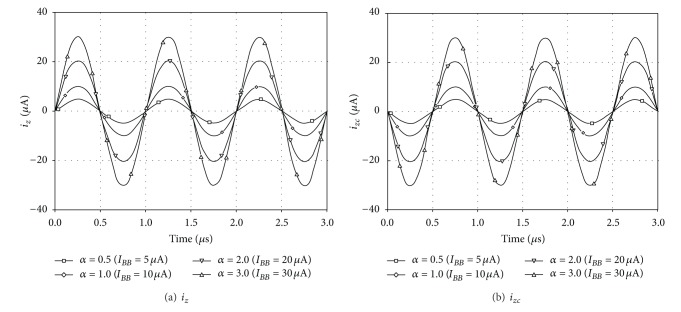
Time responses to a 10 *μ*A sinusoidal signal at 1 MHz for different values of *I*_*BB*_.

For the current transfer configuration, the DC and AC current characteristics of the output currents from *z* and *zc* terminals against the current applied to *p* terminal are shown in Figures 8 and 9, respectively. In these simulations, the bias current *I*_*BB*_ was set to the values of 5 *μ*A, 10 *μ*A, 20 *μ*A, and 30 *μ*A, respectively, while keeping *I*_*BA*_ = 10 *μ*A.  Figure 8 demonstrates the DC gain of the proposed circuit for *α* varying from 0.5 to 3. It can be observed that the circuit exhibits good linearity for input current *i*_*p*_ in the range from −12 *μ*A to 12 *μ*A.  Figure 9 is demonstrating the AC gain. The 3 dB bandwidth also proves to be constant for different AC gain values and is in a high frequency as nearly as 70 MHz. The time responses to a 1 MHz sinusoidal input with 10 *μ*A peak magnitude are also displayed in Figure 10. As shown in the figures, the current transfer characteristics with different *α* can be obtained by keeping *I*_*BA*_ and only varying *I*_*BB*_.

**Figure 11:**
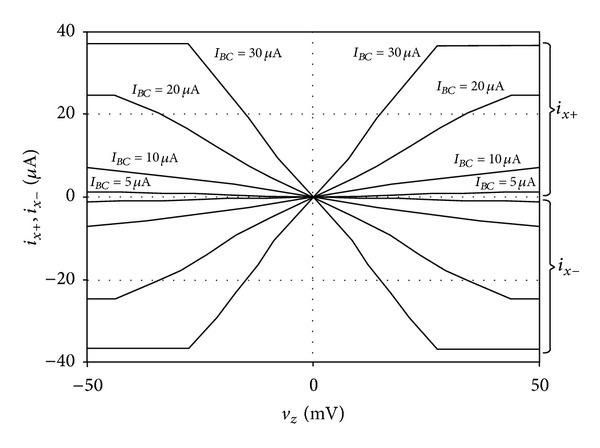
*v*_*z*_ − *i*_*x*±_ DC transfer characteristics of the proposed CG-CFTA for different values of *I*_*BC*_.

**Figure 12:**
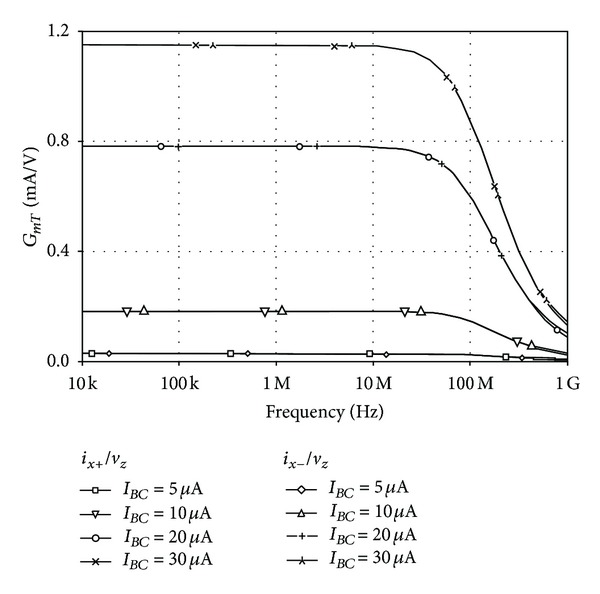
Frequency responses of *G*_*mT*_ for different values of *I*_*BC*_.

For the transconductance amplifier configuration, the DC transfer characteristics of *v*_*z*_ and *i*_*x*±_ for *I*_*BD*_ = 10 *μ*A and four different values of *I*_*BC*_ are shown in Figure 11. The plots show that the proposed circuit in Figure 6 can linearly convert the voltage signal *v*_*z*_ into current signals *i*_*x*±_ with nonlinearity of less than 1.5% for *v*_*z*_ in the ranges of −44 mV to 44 mV and −26 mV to 26 mV. These results were agreed with the prediction value from (6). For example, for the case of *I*_*BC*_ = 20 *μ*A and *I*_*BD*_ = 10 *μ*A (*G*_*mT*_≅5.67 × 10^−4^ A/V), the conversion error is about 1.32% for *v*_*z*_ = 40 mV. The frequency response of *G*_*mT*_ for different values of *I*_*BC*_ was also studied and is shown in Figure 12, where the 3 dB bandwidth of about 90 MHz is observed. The simulation results show that, under bias condition of *I*_*BA*_ = *I*_*BB*_ = *I*_*BC*_ = *I*_*BD*_ = 10 *μ*A, the total power dissipation of the CG-CFTA in Figure 6 was 0.43 mW.

## 5. Application Example

**Figure 13:**
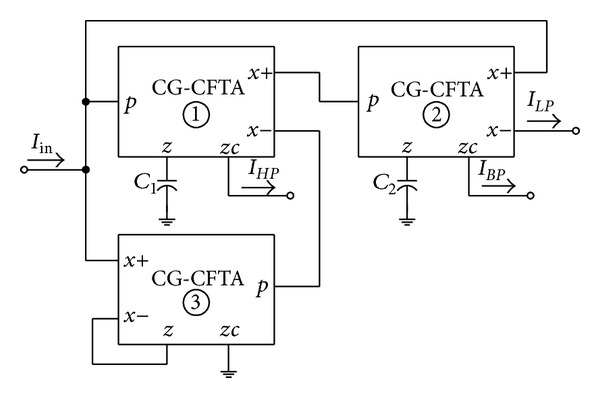
Current-mode KHN filter using CG-CFTAs.

Although the current-mode KHN biquad filter realization with ZC-CFTAs has been introduced in [20], an additional current gain block (ZC-CFTA3 and ZC-CFTA4) is required to provide an independently current tuning of the circuit quality factor (*Q*). In this section, an application example demonstrating the use of the proposed CG-CFTA in realizing current-mode KHN biquad filter is described. This demonstrates that, by employing the proposed CG-CFTA, the derived filter has fewer active components, and has the advantage of an orthogonal electronic control of the natural angular frequency (*ω*_0_) and *Q* by linearly changing the bias current ratio.  Figure 13 shows the current-mode KHN biquad realization using CG-CFTAs and grounded capacitors. Routing circuit analysis of this circuit results in low-pass (*LP*), band-pass (*BP*), and high-pass (*HP*) current transfer functions as

(10) $$\begin{array}{c} \frac{I_{LP}\left(s\right)}{I_{\text{in}}\left(s\right)}=\frac{\left(\alpha_{1}\alpha_{2}G_{mT1}G_{mT2}/C_{1}C_{2}\right)}{D\left(s\right)},\\ \frac{I_{BP}\left(s\right)}{I_{\text{in}}\left(s\right)}=-\frac{\left(\alpha_{2}/\alpha_{3}\right)\left(\alpha_{1}\alpha_{3}G_{mT1}/C_{1}\right)s}{D\left(s\right)},\\ \frac{I_{HP}\left(s\right)}{I_{\text{in}}\left(s\right)}=-\frac{\alpha_{1}s^{2}}{D\left(s\right)},\\ D\left(s\right)=s^{2}+\left(\frac{\alpha_{1}\alpha_{3}G_{mT1}}{C_{1}}\right)s+\left(\frac{\alpha_{1}\alpha_{2}G_{mT1}G_{mT2}}{C_{1}C_{2}}\right), \end{array}$$

where *α*_*i*_ and *G*_*mTi*_ are the parameters *α* and *G*_*mT*_ of *i*th CG-CFTA (*i* = 1,2, 3). The passband gains of the *LP*, *BP*, and *HP* current responses are found as *H*_*LP*_ = 1, *H*_*BP*_ = −(*α*_2_/*α*_3_), and *H*_*HP*_ = − *α*_1_, respectively.

From (10), the important parameters *ω*_0_ and *Q* of the filter in Figure 13 can be given by

(11) $$\begin{array}{c} \omega_{0}=\sqrt{\frac{\alpha_{1}\alpha_{2}G_{mT1}G_{mT2}}{C_{1}C_{2}}},\\ Q=\frac{1}{\alpha_{3}}\sqrt{\frac{\alpha_{2}G_{mT2}C_{1}}{\alpha_{1}G_{mT1}C_{2}}}. \end{array}$$

For simplicity, if we let *α* = *α*_1_ = *α*_2_ (*I*_*BA*_ = *I*_*BA*1_ = *I*_*BA*2_) and (*I*_*BB*_ = *I*_*BB*1_ = *I*_*BB*2_), *G*_*mT*_ = *G*_*mT*1_ = *G*_*mT*2_  (*I*_*BC*_ = *I*_*BC*1_ = *I*_*BC*2_), and *C* = *C*_1_ = *C*_2_, then (11) turns to

(12) $$\begin{array}{c} \omega_{0}=\left(\frac{I_{BB}I_{BC}}{I_{BA}}\right)\left(\frac{K_{T}}{C}\right),\\ Q=\frac{I_{BA3}}{I_{BB3}}. \end{array}$$

It should be noted from (12) that the filter parameters *ω*_0_ and *Q* are independently controllable. This means that the *ω*_0_ can be tuned electronically without disturbing the *Q*-value by linearly changing the current ratio *I*_*BB*_/*I*_*BA*_ and/or the bias current *I*_*BC*_. On the other hand, the value of *Q* can be tuned linearly and separately by the ratio *I*_*BA*3_/*I*_*BB*3_.

**Figure 14:**
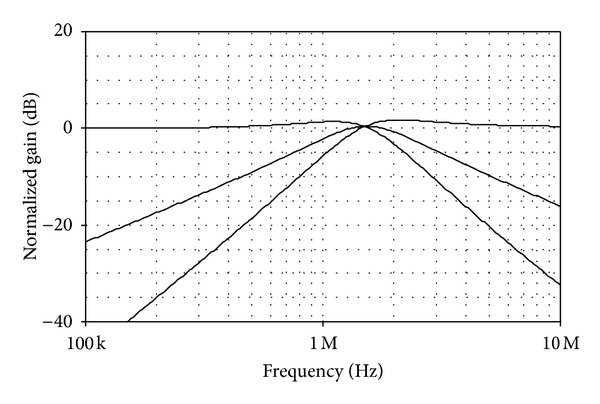
Simulated *LP*, *BP*, and *HP* current responses for the filter in Figure 13.

**Figure 15:**
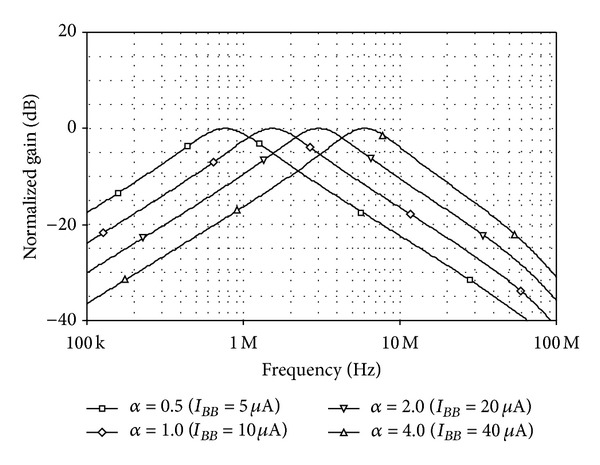
Simulated *BP* responses for different values of *I*_*BB*_.

**Figure 16:**
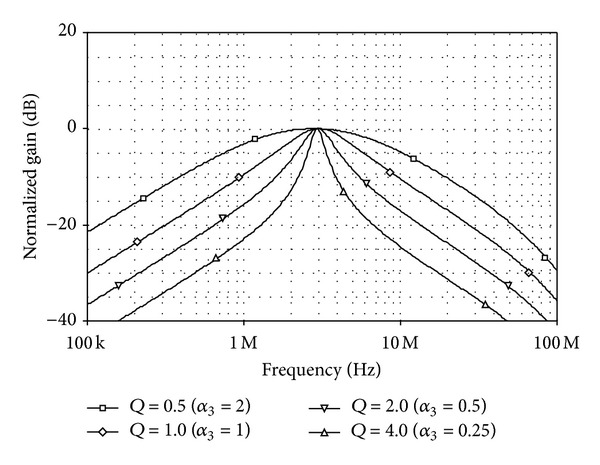
Simulated *BP* responses for different values of *I*_*BA*3_.

As an example, the following setting for the KHN filter of Figure 13 has been selected as *I*_*BAi*_ = *I*_*BBi*_ = *I*_*BCi*_ = *I*_*BDi*_ = 10 *μ*A and *C* = *C*_1_ = *C*_2_ = 20 pF, which results in *f*_0_ = *ω*_0_/2*π*≅1.51 MHz and *Q* = 1. In this setting, the total power consumption was found as 1.58 mW.  Figure 14 shows the simulated *LP*, *BP*, and *HP* current characteristics for the filter in Figure 13. To demonstrate the electronic controllability of *f*_0_, the tuning bias currents *I*_*BB*_ were varied to 5 *μ*A, 10 *μ*A, 20 *μ*A, and 40 *μ*A, respectively, while keeping *I*_*BC*_ = *I*_*BA*3_ = *I*_*BB*3_ = 10 *μ*A for *Q* = 1. The resulting *BP* responses corresponding to different bias current ratios *I*_*BB*_/*I*_*BA*_ are given in Figure 15. The *f*_0_-values obtained from the plots are approximated to 0.76 MHz, 1.51 MHz, 3.02 MHz and 6.02 MHz, respectively.  Figure 16 also shows the simulated *BP* responses with *Q*-tuning (i.e., *Q* = 0.5, 1, 2, and 4). In this case, the bias currents were chosen as *I*_*BA*_ = *I*_*BB*3_ = *I*_*BC*_ = *I*_*BD*_ = 10 *μ*A, *I*_*BB*_ = 20 *μ*A, and *I*_*BA*3_ = 5 *μ*A, 10 *μ*A, 20 *μ*A, 40 *μ*A, respectively, resulting in *f*_0_≅3.02 MHz.

## 6. Conclusion 

In this paper, we have presented the design of a generalized current follower transconductance amplifier using only transconductance cells. We have named it as the controlled-gain current follower transconductance amplifier (CG-CFTA), since its current transfer ratios (*i*_*z*_/*i*_*p*_, *i*_*zc*_/*i*_*p*_ and *i*_*x*±_/*i*_*z*_) can be varied linearly by external bias currents. The circuit has the features of low voltage supply, low power consumption, large bandwidth, and convenient for realization in CMOS technology. Simulation results confirm the qualification performances of the circuit. To illustrate the design possibility provided by the newly defined circuit, the current-mode KHN-equivalent biquad filter is constructed and simulated.
